# Semaphorin4A causes loss of mature oligodendrocytes and demyelination in vivo

**DOI:** 10.1186/s12974-019-1420-9

**Published:** 2019-02-08

**Authors:** Brian Chiou, Elizabeth Neely, Asha Kallianpur, James R. Connor

**Affiliations:** 1 Department of Neurosurgery, Penn State University College of Medicine, 500 University Drive, Hershey, PA 17033 USA; 2 Department of Genomic Medicine, Cleveland Clinic/Lerner Research Institute, Cleveland, OH USA; 3 Department of Molecular Medicine, Cleveland Clinic Lerner College of Medicine, Case Western Reserve University, Cleveland, OH USA

**Keywords:** Sema4A, Demyelination, HIV, Apoptosis, Oligodendrocyte, White matter

## Abstract

**Background:**

Inappropriate contact between the immune system and the central nervous system is thought to be a cause of demyelination. We previously reported the ability of the class IV semaphorin, Semaphorin4A (Sema4A), to induce apoptosis in human oligodendrocytes; however, these results have yet to be translated to an in vivo setting. Importantly, HIV-associated neurocognitive disorder remains a significant complication for patients on combined anti-retroviral therapy, with white matter damage seen on MRI.

**Methods:**

Human cerebrospinal fluid and serum was assayed for Sema4A using a Sema4A-specific ELISA. Wild-type mice were injected with Sema4A via stereotaxic infusion. Data was assessed for significance using unpaired *t* tests, comparing the corpus callosum of PBS-injected mice versus Sema4A-injected mice.

**Results:**

Here, we demonstrate elevated levels of Sema4A in the cerebrospinal fluid and serum of people with HIV infection. Furthermore, we demonstrate that direct injection of Sema4A into the corpus callosum of mice results in loss of myelin architecture and decreased myelin, concomitant with apoptosis of mature myelinating oligodendrocytes. Sema4A injection also causes increased activation of microglia.

**Conclusions:**

Taken together, our data further establish Sema4A as a potentially significant mediator of demyelinating diseases and a direct connection between the immune system and oligodendrocytes.

## Background

Demyelination in the central nervous system (CNS) has long been thought to be a result of inappropriate immune activation, leading to targeted destruction of the myelin sheath, as well as a loss of mature myelinating oligodendrocytes [1, 2]. Though the brain is typically segregated from the peripheral immune system, trafficking of immune cells or immune trophic factors across the blood-brain barrier may lead to inappropriate interactions between immune cells, oligodendrocytes, and the myelin sheath. Previous studies by our laboratory have demonstrated that exposure to Semaphorin4A (Sema4A), an immune trophic factor required for T cell activation [3], is implicated in the death of oligodendrocytes [4, 5]. Furthermore, Sema4A has been shown to be involved in experimental autoimmune encephalomyelitis (EAE) mouse model pathology [3, 6].

Classically, Sema4A has been described as a neuronal axon guidance cue during development [7]. However, recent literature has demonstrated the importance of Sema4A in immune-mediated diseases such as rheumatoid arthritis [8] and asthma [9]. Importantly, Sema4A has been shown to be expressed by activated microglia [4], dendritic cells [10], activated T cells [11], and germinal center B cells [12]. Previous studies have also demonstrated that Sema4A aids in T cell maturation by binding to the T cell immunoglobulin and mucin domain 2 (Tim-2) receptor in rodents [3] and to Tim-1 in humans [5]. We have previously demonstrated that Tim-1 and Tim-2 are present on oligodendrocytes in humans and rodents, respectively, and that this receptor binds to and mediates the uptake of H-ferritin [5, 13–15].

HIV-associated neurocognitive disorder (HAND) remains a significant complication among individuals on combination antiretroviral therapy (cART) for HIV infection [16]. By current diagnostic criteria, up to 50% of all HIV-seropositive (HIV+) individuals experience some form of HAND [17], which is marked by white matter damage in the brain and neurocognitive impairment (NCI) [18]. NCI in people with HIV has been associated with higher volumes of abnormal white matter, as well as decreased volumes of total white matter [18, 19]. In addition to an infrequently seen, acute-onset immune reconstitution inflammatory syndrome (IRIS) with white matter abnormalities on neuroimaging, which can occur in the central nervous system upon initiation of cART, white matter alterations have also been associated with greater degrees of CD4+ T cell recovery on cART, indicating a role for neuroinflammation in the pathogenesis of these changes [20]. The mechanisms for white matter damage related to HIV infection have yet to be elucidated. We hypothesized that the white matter abnormalities seen in HIV+ persons and in patients with multiple sclerosis (MS) could be at least in part mediated by the direct interaction between Sema4A and Tim-1 receptors on human oligodendrocytes [5]. This hypothesis is supported by a number of clinical studies, which have suggested that high circulating Sema4A levels are associated with resistance to some types of treatment in patients with MS and may be useful for risk stratification [10, 21].

Though we have previously reported Sema4A to have a direct cytotoxic effect on mature oligodendrocytes in vitro [5], the ability of Sema4A to cause cell death and demyelination in vivo has yet to be explored. In this study, we examined the effects of in vivo Sema4A intracranial injection on white matter in the adult mouse brain. Here, we show that direct injection of Sema4A into the corpus callosum of adult mice can lead to loss of white matter, as well as to oligodendrocyte cell death in the brain.

## Materials and methods

### Intracranial injection and brain isolation

A total of 10 6-month-old, wild-type 129/C57Bl/6J mice were sorted into 2 groups: a phosphate-buffered saline (PBS) injection control group (*n* = 5) and a recombinant Sema4A-Fc (AcroBiosystems) injection group (*n* = 5). The mice were anesthetized using a 5% isoflurane mixture and maintained under anesthesia using a 1–2% isoflurane mixture. Next, the heads of mice were shaved, and povidone-iodine was used to disinfect the area immediately prior to surgery. Lubrication was applied to the cornea, and mice were mounted upon the stereotaxic frame. After immobilization via ear bars, a small incision was made in the skin, exposing the skull. A burr hole was drilled, and either a 1 μg/mL Sema4A-PBS solution or an equivalent volume of PBS alone (5 μL) was injected into the corpus callosum (coordinates 0.5 mm anterior, 1.0 mm lateral, and 2.5 mm deep, relative to bregma) using a 22-gauge needle and syringe (Hamilton). Following injection, bone wax (Harvard Apparatus) was applied to the skull to prevent leakage and the skin was sutured. Mice were taken off isoflurane, warmed in a 37°C environment, and allowed to wake before transport. Mice were monitored daily until 7 days post-surgery, when they were anesthetized with a ketamine/xylazine cocktail and transcardially perfused, first with Ringer’s solution and then with 4% paraformaldehyde. Brains were removed and put into a 4% paraformaldehyde solution overnight, followed by 0.1 M PBS solution the next day. All procedures were conducted in accordance with the NIH Guide for the Care and Use of Laboratory Animals, formally approved by the Pennsylvania State University College of Medicine International Animal Care and Use Committee under Protocol #47464, and reported in accordance with Animal Research: Reporting In Vivo Experiments.

### Immunohistochemistry

One day prior to paraffin embedding, the brains were transferred to a 70% ethanol solution. Brains were paraffin-embedded and 5 μm coronal sections were taken and mounted upon standard microscopy slides. We have previously described our immunohistochemistry methods [22, 23]. Briefly, slides were deparaffinized in xylenes and rehydrated in a standard ethanol gradient. Antigen retrieval was performed using a 10 mM citrate buffer solution (pH 6.0). Slides were blocked in a 3% H_2_O_2_/methanol solution, rinsed in 1× PBS, and blocked for 1 h in a 2% milk/PBS solution. The slides were then incubated overnight in primary antibody at 4°C using anti-cleaved caspase-3 (Cell Signaling, 9661, 1:200), anti-MBP (Abcam, ab7349, 1:200), anti-Olig2 (Abcam, ab109186, 1:200), anti-CC1 (Calbiochem, OP80, 1:100), anti-CNPase (Abcam, ab6319, 1:200), anti-Iba1 (Wako, 019-19741, 1:200), anti-CD3 (Abcam, ab5690, 1:200), or anti-GFAP (Agilent, Z0334, 1:1000). The following day, the slides were washed in a 1× PBS solution and incubated with species-specific secondary antibodies using the ABC Vectastain kit (1:200, Vector Labs) according to the manufacturer’s protocol or species-specific AlexaFluor488 and AlexaFluor555 secondary antibodies. The slides were then treated with diaminobenzidine (DAB) to visualize the secondary antibodies, using nickel chloride to intensify DAB staining. Following staining, slides were dehydrated, mounted, coverslipped, and allowed to dry overnight. The terminal deoxynucleotidyl transferase (TdT)-mediated dUTP nick-end labeling (TUNEL) assay was performed using the DeadEnd Fluorometric TUNEL System (Promega, G3250) to detect apoptosis according to the manufacturer’s given protocol after rehydration of formalin-fixed, paraffin-embedded tissue.

For Luxol fast blue staining, paraffin-embedded slides were deparaffinized and rehydrated in an ethanol gradient up to 95% ethanol. Slides were then stained in a 0.1% Luxol fast blue solution overnight at 56°C. The following day, slides were rinsed in dH_2_O and differentiated in a 0.05% lithium carbonate solution. Subsequently, slides were stained in a 0.1% cresyl violet solution for 30 s and differentiated in 95% ethanol. Slides were then dehydrated, mounted, coverslipped, and allowed to dry overnight.

For image quantification, images of both halves of the corpus callosum were taken on a standard light microscope at 20× and analyzed on ImageJ. A region of interest (ROI) was created by outlining the corpus callosum (dotted lines), and percent area fraction was measured in that ROI. Percent area fraction was used to measure immunostaining density, reflecting the relative amounts of staining in each corpus callosum half. Researchers were blinded to patient clinical status and HIV serostatus, as well as for creation of the ROI and for the duration of the analyses. Measurements were reported as the ratio of injected corpus callosum signal to uninjected corpus callosum signal. The brains from all five mice in each group were stained, and the percent area fraction quantified.

### Protein assays

Cerebrospinal fluid (CSF) and serum samples (*n* = 50) were obtained from HIV+ adults who were previously recruited to the multicenter HIV Neurobehavioral Research Center (HNRC) at the University of California, San Diego. HIV+ study participants provided written informed consent for lumbar puncture, medical records review, and comprehensive neuropsychiatric testing [19].

Sema4A protein content in the CSF and serum was quantified using a Sema4A-specific ELISA (Biomatik), following the manufacturer’s protocol. Positive controls used for this ELISA were a recombinant Sema4A-Fc protein (AcroBiosystems), as well as CSF samples used in our previous study [5] that showed positive signal. Negative CSF used in this assay came from HIV-seronegative patients without demyelinating disease (CSF *n* = 19, serum *n* = 10).

### Statistical analysis

Statistical analyses were performed using GraphPad Prism 4 software (GraphPad Software, Inc.). Data were collected from five independent biological replicates, averaged, and expressed as the mean ± SD. Unpaired *t* tests were used to compare the PBS- and Sema4A-injected groups to evaluate statistical significance. *p* values < 0.05 were considered significant.

## Results

### Sema4A is elevated in the CSF and serum of HIV+ individuals

**Fig. 1:**
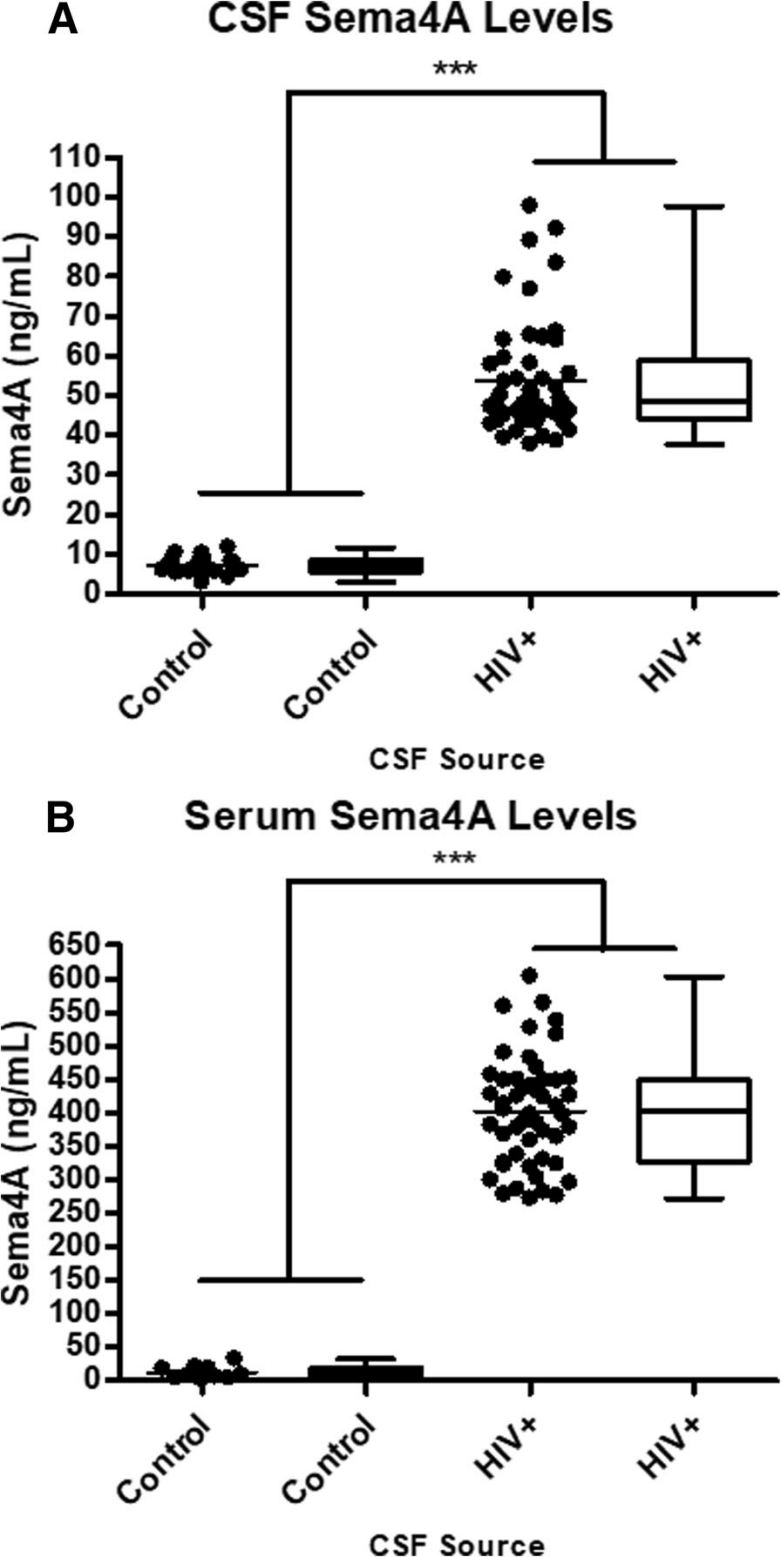
Elevated levels of Sema4A in the CSF of HIV-infected individuals. **a** CSF from HIV+ individuals displays a significantly higher level of Sema4A levels (53.72 ± 14.37 ng/mL, *n* = 50) relative to HIV-negative, non-demyelinated control patients (7.22 ± 2.28 ng/mL, *n* = 19). **b** Serum values of Sema4A from HIV+ individuals (401.92 ± 81.73 ng/mL, *n* = 50) is significantly elevated relative to HIV-negative, non-demyelinated control patients (12.38 ± 9.78 ng/mL, *n* = 10). Though the data are the same, scatterplot lines represent mean values, whereas the box and whisker plots represent the medians and interquartile ranges. Statistical significance was evaluated using the Mann-Whitney test; ***p < 0.0001

The levels of Sema4A were measured in 50 HIV+ individuals who consented to have lumbar punctures and blood measurements as part of previously conducted studies at the University of California-San Diego HIV Neurobehavioral Research Center (HNRC). Using a Sema4A-specific ELISA, we determined that Sema4A levels are higher in individuals with HIV infection (53.72 ± 14.37 ng/mL) compared with HIV-seronegative, control individuals without known demyelinating disease. (7.22 ± 2.28 ng/mL; *p* < 0.001). Furthermore, we demonstrated that serum Sema4A levels are significantly elevated in HIV+ individuals (401.92 ± 81.73 ng/mL) compared with HIV-seronegative individuals (12.38 ± 9.78 ng/mL; *p* < 0.001) (Fig. 1).

### Sema4A causes demyelination

**Fig. 2:**
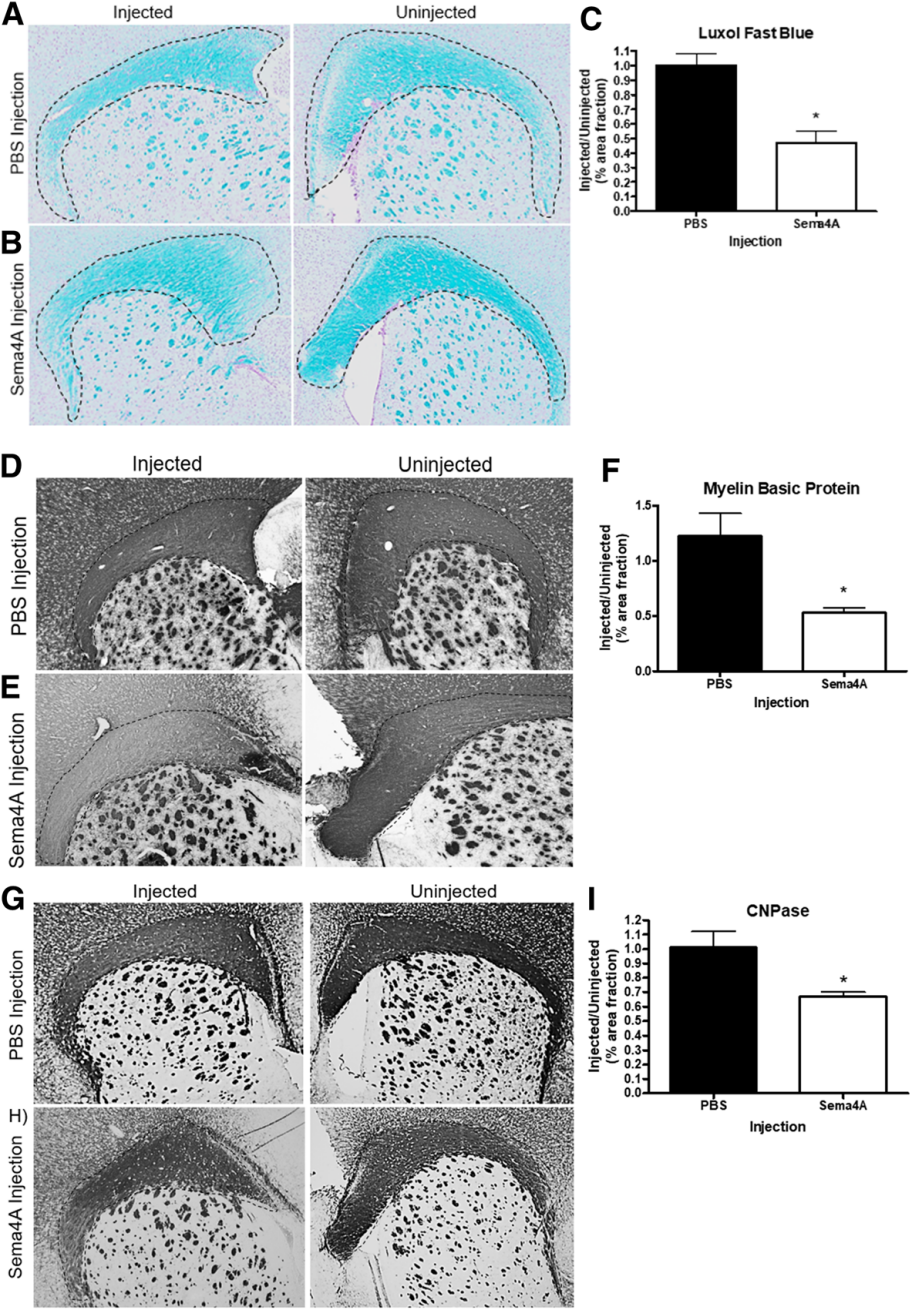
Sema4A causes demyelination of the corpus callosum. Sema4A (1 μg/mL) was injected into the corpus callosum, brain slices were obtained 7 days post-surgery, and tissue slices were stained with Luxol fast blue (**a**, **b**, **c**), myelin basic protein (**d**, **e**, **f**), or CNPase (**g**, **h**, **i**) to visualize the myelin sheath. **a** Equivolume PBS injection does not cause demyelination. **b** Sema4A injection causes gross demyelination and loss of architecture. **c** Quantification of decreased overall myelination (54%) in the outlined region of interest. **d** PBS injection does not affect MBP staining intensity. **e** Sema4A injection results in decreased levels of MBP staining. **f** MBP staining is decreased by 48% after Sema4A injection. **g** CNPase staining is unaffected by PBS injection. **h** Sema4A treatment decreases total CNPase staining by 34% (**i**). PBS and Sema4A groups each had *n* = 5. Unpaired *t* tests were used to evaluate differences between PBS and Sema4A groups; *p < 0.05

Previously, we established that both purified and recombinant Sema4A is cytotoxic to oligodendrocytes in vitro*,* in both rodent and human primary oligodendrocyte cells [4, 5]. To investigate the effects of Sema4A in vivo, we performed stereotaxic injections of recombinant Sema4A into the corpus callosum of wild-type 129/C57Bl/6J mice. Following infusion of PBS, there was no demyelination evident on the injected side compared to the uninjected side (Fig. 2a). However, following Sema4A injection, there was clear evidence of demyelination (Fig. 2b) when compared to the uninjected side. By quantifying the myelination in separate regions of the corpus callosum by Luxol fast blue staining, we observed a 54% decrease in myelin content (*p* = 0.011) associated with Sema4A injection (Fig. 2c) when compared to PBS injection.

To further characterize demyelination after Sema4A injection, we assessed levels of myelin basic protein (MBP) and 2′,3′-cyclic nucleotide 3′-phosphodiesterase (CNPase), both major contributors to the integrity of myelin architecture. Similar to Luxol Fast Blue staining, injection of PBS was not associated with any decrease in myelination compared to the uninjected side (Fig. 2d). However, injection of Sema4A (Fig. 2e) caused a 48% decrease in MBP staining compared to the uninjected side (Fig. 2f, *p* = 0.03). Similarly, when we stained for CNPase, a marker for early lineage oligodendrocytes and developing myelin, PBS injection had no effect on CNPase levels (Fig. 2g), while Sema4A injection (Fig. 2h) caused a 34% decrease in CNPase content (Fig. 2i, *p* = 0.04). We did not observe any demyelination of other white matter structures, such as the deep cerebellar white matter or anterior commissure (data not shown).

### Sema4A causes loss of mature oligodendrocytes in vivo

**Fig. 3:**
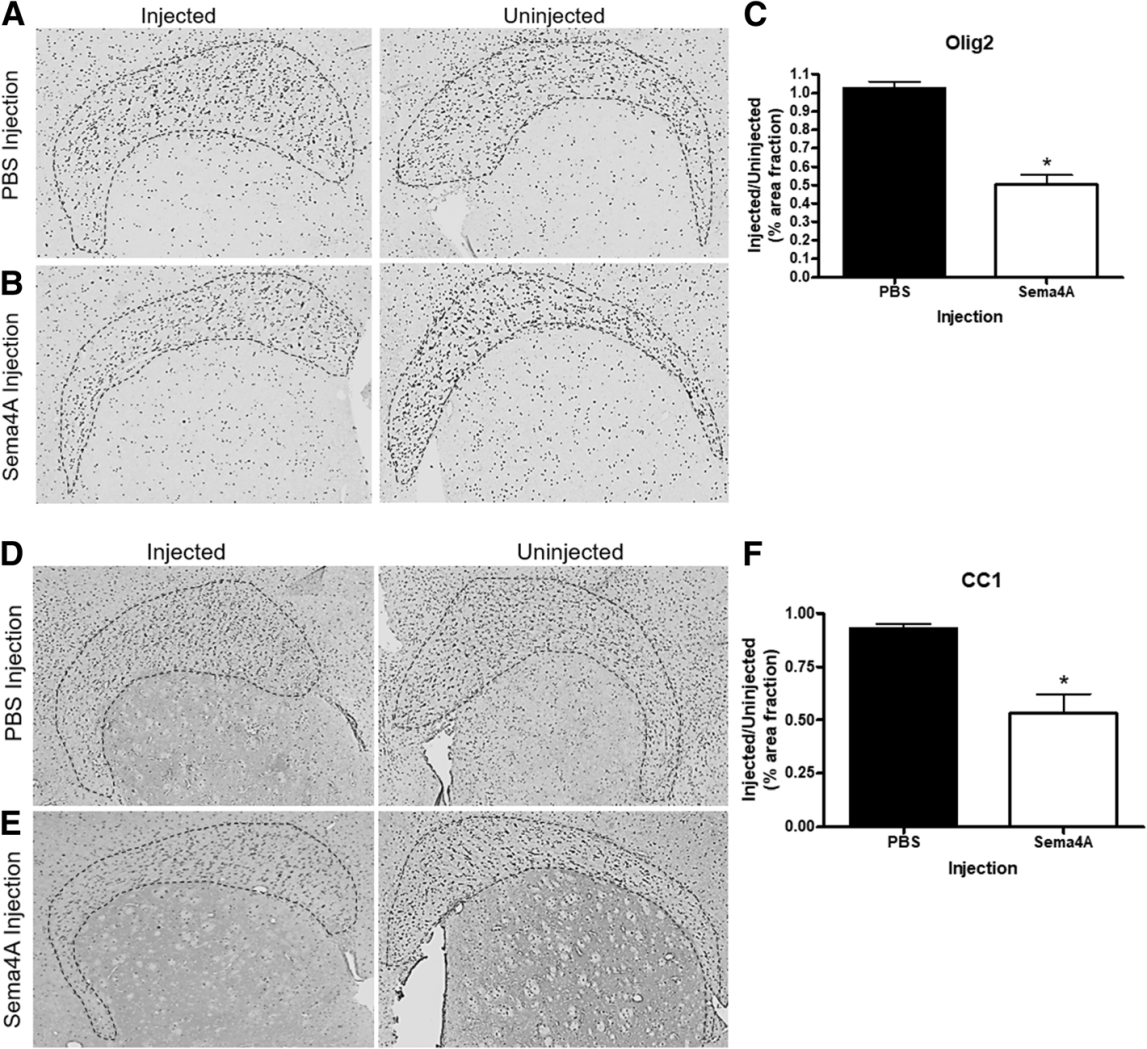
Sema4A treatment causes loss of mature myelinating oligodendrocytes. The brains treated with PBS or Sema4A were sliced and stained for Olig2, a pan-oligodendrocyte marker, or CC1, a mature myelinating oligodendrocyte marker. **a** PBS injection causes no loss in total oligodendrocyte numbers. **b** Sema4A injection causes a decrease in total oligodendrocytes. **c** 50% oligodendrocyte loss is observed after Sema4A treatment. **d** PBS injection causes no loss in mature myelinating oligodendrocytes. **e** Sema4A injection causes a decrease of mature myelinating oligodendrocytes by 47% (**f**). PBS and Sema4A groups each had *n* = 5. Unpaired *t* tests were used to compare PBS and Sema4A groups; *p < 0.05

We next assessed whether Sema4A treatment causes loss of oligodendrocytes in vivo*,* by measuring levels of Olig2, a pan-oligodendrocyte marker, and CC1, a marker for mature myelinating oligodendrocytes. Following PBS injection, there was no observable difference in oligodendrocyte numbers in the corpus callosum (Fig. 3a). However, when Sema4A was injected, there was a 50% decrease in Olig2 staining (Fig. 3b, c, *p* = 0.001). Similarly, we observed no change in CC1 staining after PBS injection (Fig. 3d), but we detected a 47% decrease in the Sema4A-injected condition (Fig. 3e, Fig. 3f, *p* = 0.01).

**Fig. 4:**
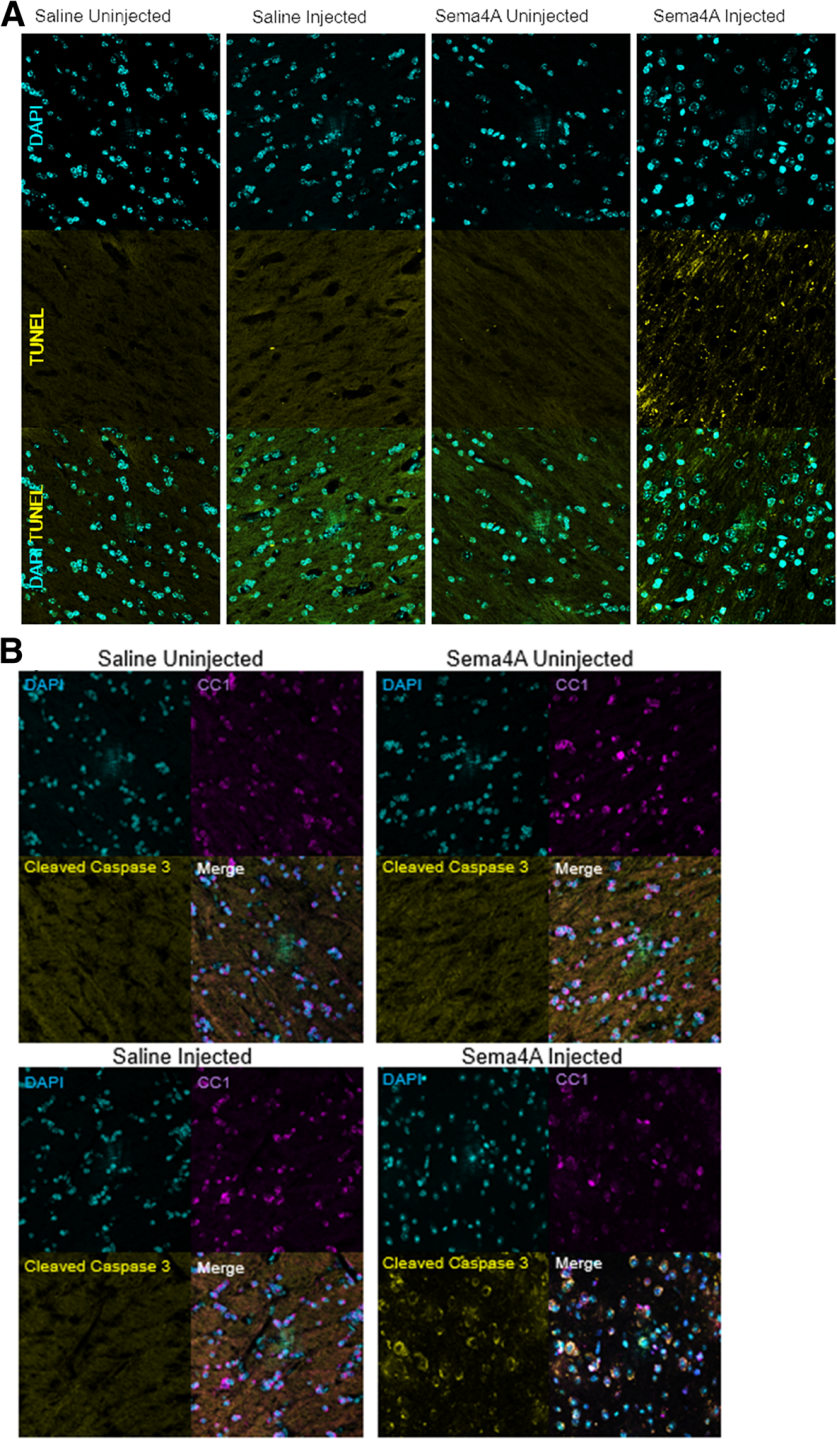
Sema4A causes apoptosis in mature myelinating oligodendrocytes in vivo. **a** Infusion of Sema4A into the corpus callosum results in increased TUNEL staining, indicating increased levels of apoptosis, relative to the uninjected regions as well as the PBS control. **b** Sema4A treatment results in increased levels of cleaved caspase-3 relative to the uninjected and PBS-injected regions

To evaluate the mechanism of cell death in the injected white matter, we performed TUNEL staining, hypothesizing that Sema4A causes an induction of apoptosis, similar to what was seen in previous in vitro studies [5] (Fig. 4a). Consistent with our results shown in Fig. 3, we observed evidence of increased DNA degradation by TUNEL assay in the white matter of Sema4A-injected mice, relative to PBS-injected mice. Furthermore, we demonstrated that cleaved executioner caspase 3, a marker for late apoptosis, is increased in CC1-positive cells in the white matter (Fig. 4b). However, uninjected white matter and PBS-injected mice did not display this increase in cleaved caspase 3.

### Sema4A causes microglial activation

**Fig. 5:**
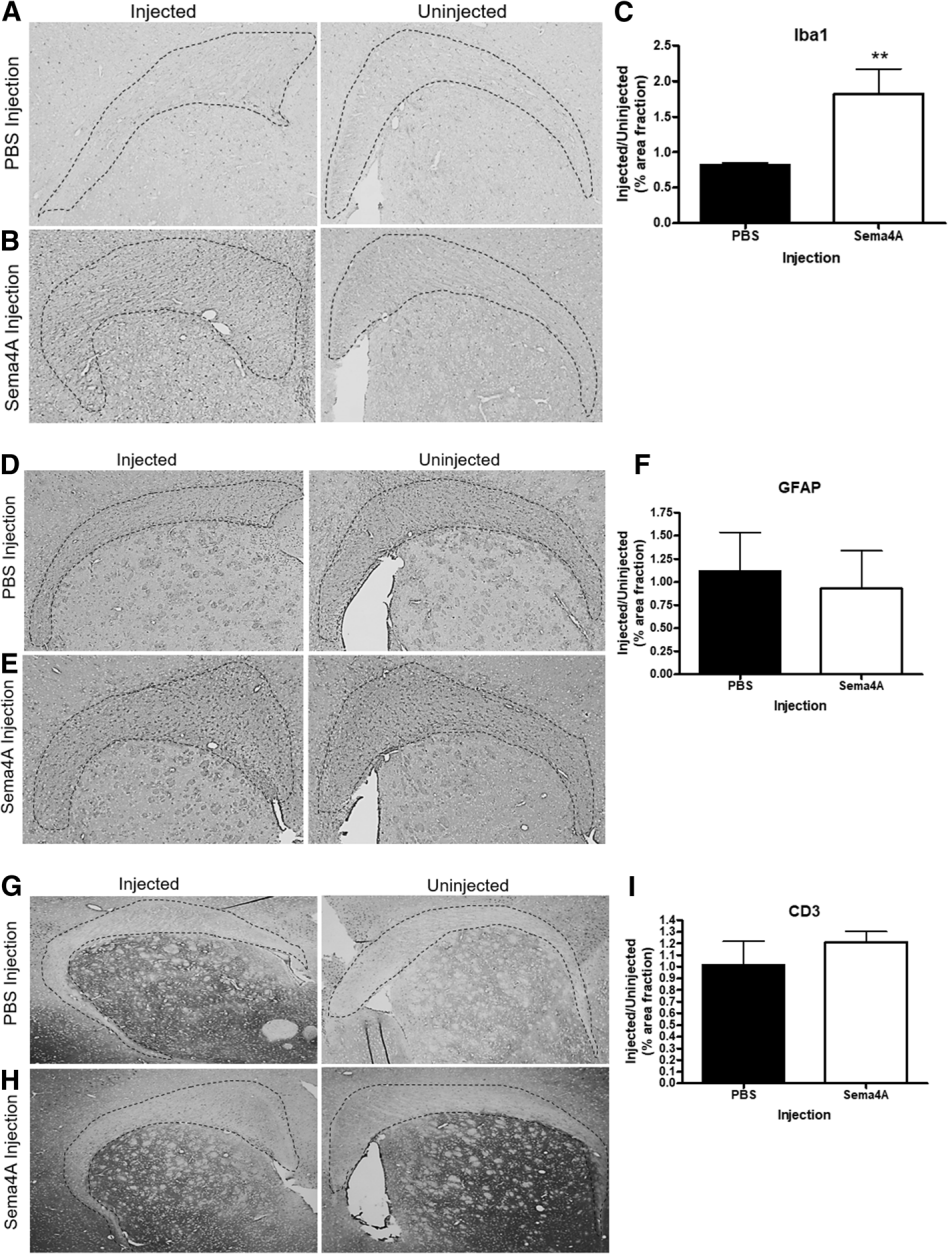
Sema4A causes an increase in locally activated microglia. The brain tissue slices were stained for Iba1, a microglial marker, GFAP, an astrocytic marker, and CD3, a pan-lymphocyte marker. **a** PBS injection does not affect Iba1 signal. **b** Sema4A injection causes an increase in Iba1 signal of 223% (**c**). Both PBS (**d**) and Sema4A (**e**) had no effect on astrocyte numbers, as indicated by non-significant differences in GFAP staining. Furthermore, CD3 signal was not increased in the PBS (**g**) or Sema4A (**h**) injection groups. PBS and Sema4A groups had *n* = 5. Unpaired *t* tests were used to compare PBS and Sema4A groups; *p < 0.05

We next examined if local microglial activation was affected by Sema4A injection by assaying the levels of Iba1 staining in corpus callosum. In our PBS-injection model, we observed no noticeable alterations in microglial Iba1 staining either in the injected or uninjected halves of the corpus callosum (Fig. 5a). There was, however, a significant increase (223%) in the amount of Iba1 expression following Sema4A injection (Fig. 5b, c, *p* = 0.04). An increase in reactive astrocytes (astrogliosis) is commonly observed in the event of significant CNS challenges, including trauma. To that end, we measured glial fibrillary acidic protein (GFAP), a marker for astrocytes, after injection with either PBS or Sema4A. In our injection model, injection of neither PBS (Fig. 5d) nor Sema4A (Fig. 5e) was associated with significant alterations in GFAP signal (Fig. 5f). Previous literature has demonstrated that Sema4A is highly expressed by activated T cells in both rodents and humans [3, 10, 24, 25]. Therefore, we assessed whether Sema4A treatment also influences the numbers of infiltrating T lymphocytes, thereby contributing to demyelination, by staining for CD3, a pan-lymphocyte marker. No significant difference was observed in total CD3 staining in the corpus callosum in either the PBS-injection (Fig. 5g) and Sema4A-injection (Fig. 5h) models (Fig. 5i).

## Discussion

Damage to white matter in the brain, which consists largely of myelinated nerve fibers, may result from either immune-mediated damage to oligodendrocytes or the myelin sheath, a failure of oligodendrocyte-mediated remyelination after injury, or from a combination of these processes [26, 27]. Previous studies by our laboratory and others have suggested that Sema4A could be a significant and direct contributor to oligodendrocyte cell loss and subsequent demyelination or white matter damage in diseases such as MS [6, 10, 21], HIV infection [5], neuroinflammation [28], and even cerebral malaria [29]. None of the previous studies provided direct in vivo evidence of a pathogenic role for Sema4A, however. In this study, we demonstrate significantly elevated levels of CSF Sema4A in a substantial sample of HIV+ persons and extend our previous pre-clinical and in vitro findings regarding Sema4A-mediated oligodendrocyte cell death to an in vivo model. We observed significant demyelination as a result of intracranial Sema4A injection of the corpus callosum, concomitant with increased apoptosis of CC1-expressing (mature) myelinating oligodendrocytes and an increase in local microglial activation. Importantly, we did not observe any cell death or demyelination outside of the corpus callosum.

Translation of our Sema4A-related in vitro findings of oligodendrocyte cytotoxicity to an in vivo model is a crucial step forward in establishing Sema4A as a potentially important factor involved in the pathogenesis of demyelinating diseases. Moreover, we provide additional evidence in a larger sample of HIV+ individuals corroborating the observation from our initial pilot study that Sema4A levels are elevated in the CSF in individuals with HIV infection [5]. White matter alterations seen in HIV+ individuals are often detected early in the disease course [30] and may occur partly as a result of acute inflammatory damage during untreated HIV infection, and partly due to CD4+ T cell recovery during cART, with inappropriate contact between the immune system and the CNS. While demyelination and elevated levels of myelin basic protein in CSF were reported in people with HIV/AIDS in the early 1990s, these findings were generally confined to patients with the AIDS Dementia Complex (ADC), progressive multifocal leukoencephalopathy (PML) caused by the neurotropic JC virus, or other opportunistic infections of the CNS [31, 32]. Although PML occasionally still occurs in HIV+ individuals despite cART and virus suppression, ADC is now rare, occurring primarily in late, untreated HIV disease. The reasons for persistent white matter injury in the cART era, which correlates with symptomatic HAND as well as longer duration of HIV infection, are therefore unclear [33, 34]. Accumulating evidence suggests that white matter injury during HIV infection in individuals on cART may be multifactorial, with contributions from immune-mediated damage, blood-brain barrier disruption, HIV viral proteins, and antiretroviral drug effects on oligodendrocyte processes [16].

In MS, the prototypical demyelinating disease of the CNS, Sema4A levels have been shown to be elevated in serum [10]. Elevation of Sema4A in the CSF as well as serum in both MS [5] and HIV infection suggests a role for Sema4A in the etiology of demyelination and other types of white matter damage in human neuroinflammatory diseases that warrants further investigation. Intriguingly, HIV has been associated with a markedly reduced incidence of MS, possibly due to HIV-mediated immunosuppression or to ameliorating effects of HIV and/or antiretroviral drugs on MS pathogenesis [35]. The source of soluble Sema4A, found normally and/or during inflammation within the brain has yet to be elucidated, although it is known to be expressed by activated microglia [4], activated T and B cells [3, 12], and dendritic cells [36]. We examined the brain following Sema4A injections for the presence of increased numbers of CD3+ T cells, microglia, and astrocytes. While astrocyte and lymphocyte numbers were unaffected by injection of Sema4A into the corpus callosum, the number of microglial cells was significantly increased in the injected region. Interestingly, a previous study by our laboratory found colocalization of CD11b, a marker of microglia and macrophages, and Sema4A in human white matter [4]. Furthermore, that study also found colocalization of the pan-T cell marker CD3 and Sema4A; histological analyses demonstrated that Sema4A is expressed by microglia and T cells, suggesting that these cells could also be a source of Sema4A, contributing to demyelination [4]. Further studies are needed to understand how Sema4A expressed by these cells is rendered soluble. Sema4D, a related member of the semaphorin family, has been shown to be proteolytically cleaved from the cell membrane and thereby rendered soluble [28, 37]. A similar mechanism may exist for Sema4A.

Our current findings, together with previous studies, implicate elevated soluble Sema4A levels as a direct mediator of the death of mature myelinating oligodendrocytes and demyelination. However, it is currently not known how Sema4A gets into the CSF or if Sema4A can cross the blood-brain barrier in its native form. We previously demonstrated that Sema4A in the CSF of persons with demyelinating disease is biologically active, causing oligodendrocyte cell death [5]. The CSF is considered to be in dynamic equilibrium with the extracellular fluid in the brain, and thus the model we have chosen of direct intracranial injection of Sema4A approximates the clinical situation. In our current study, we treated mice with a single dose of 1 μg/mL recombinant Sema4A protein. In a demyelinating disease state, the Sema4A CSF concentration may range from 50 ng/mL (Fig. 1) to as high as 120 ng/mL [5]. The higher concentration of Sema4A used in this study was selected because we were using an acute single bolus injection model rather than a chronic low-dose infusion in which the cells are potentially incubated in the toxin for hours to days. The data herein establish that exposure to Sema4A is toxic to oligodendrocytes in vivo as it is in vitro.

## Conclusions

Overall, the data presented in this study further illustrate a role for Sema4A-oligodendrocyte interaction in the etiology of demyelinating diseases by showing direct, adverse in vivo effects of the protein on oligodendrocytes and myelin homeostasis. These findings continue to provide compelling evidence for Sema4A as a clinical link between the immune system and damage to white matter in the brain in demyelinating disorders. The degree of contribution of Sema4A produced by trafficking immune cells and/or resident cells in the brain, such as microglia, to the pathogenesis of demyelinating disorders requires further study.

## Abbreviations

cART: Combined anti-retroviral therapy
CNPase: 2′-3′-cyclic nucleotide 3′-phosphodiesterase
CNS: Central nervous system
CSF: Cerebrospinal fluid
DAB: Diaminobenzidine
EAE: Experimental autoimmune encephalomyelitis
ELISA: Enzyme-linked immunosorbent assay
GFAP: Glial fibrillary acidic protein
HAND: HIV-associated neurocognitive disorder
HIV: Human immunodeficiency virus
IRIS: Immune reconstitution inflammatory syndrome
LFB: Luxol fast blue
MBP: Myelin basic protein
MS: Multiple sclerosis
NCI: Neurocognitive impairment
PBS: Phosphate-buffered saline
Sema4A: Semaphorin4A
Tim-2: T cell immunoglobulin and mucin domain receptor
TUNEL: Terminal deoxynucleotidyl transferase (TdT)-mediated dUTP nick-end labeling

## References

[1] Noseworthy JH, Lucchinetti C, Rodriguez M, Weinshenker BG (2000). Multiple sclerosis. N Engl J Med.

[2] Love S (2006). Demyelinating diseases. J Clin Pathol.

[3] Kumanogoh A, Marukawa S, Suzuki K, Takegahara N, Watanabe C (2002). Ch’ng E, et al. Class IV semaphorin Sema4A enhances T-cell activation and interacts with Tim-2. Nature.

[4] Leitner DF, Todorich B, Zhang X, Connor JR (2015). Semaphorin4A is cytotoxic to oligodendrocytes and is elevated in microglia and multiple sclerosis. ASN Neuro.

[5] Chiou B, Lucassen E, Sather M, Kallianpur A, Connor J (2018). Semaphorin4A and H-ferritin utilize Tim-1 on human oligodendrocytes: a novel neuro-immune axis. Glia..

[6] Koda T, Okuno T, Takata K, Honorat JA, Kinoshita M, Tada S (2014). Sema4A inhibits the therapeutic effect of IFN-β in EAE. J Neuroimmunol.

[7] Yukawa K, Tanaka T, Bai T, Ueyama T, Owada-Makabe K, Tsubota Y (2005). Semaphorin 4A induces growth cone collapse of hippocampal neurons in a Rho/Rho-kinase-dependent manner. Int J Mol Med.

[8] Wang L, Song G, Zheng Y, Tan W, Pan J, Zhao Y (2015). Expression of Semaphorin 4A and its potential role in rheumatoid arthritis. Arthritis Res. Ther. BioMed Central.

[9] Mogie G, Shanks K, Nkyimbeng-Takwi E, Smith E, Davila E, Lipsky M (2013). Neuroimmune semaphorin 4A as a drug and drug target for asthma. Int Immunopharmacol.

[10] Nakatsuji Y, Okuno T, Moriya M, Sugimoto T, Kinoshita M, Takamatsu H (2012). Elevation of Sema4A implicates Th cell skewing and the efficacy of IFN-β therapy in multiple sclerosis. J Immunol.

[11] Toyofuku T, Yabuki M, Kamei J, Kamei M, Makino N, Kumanogoh A (2007). Semaphorin-4A, an activator for T-cell-mediated immunity, suppresses angiogenesis via Plexin-D1. EMBO J.

[12] Linder GE, Chuntova PD, McLelland BT, Añó L, Obodo UC, Crider NJ (2013). Semaphorin 4A is dynamically regulated during thymocyte development in mice. Cell Immunol.

[13] Todorich B, Zhang X, Slagle-Webb B, Seaman WE, Connor JR (2008). Tim-2 is the receptor for H-ferritin on oligodendrocytes. J Neurochem.

[14] Chiou B, Neal EH, Bowman AB, Lippmann ES, Simpson IA, Connor JR. Endothelial cells are critical regulators of iron transport in a model of the human blood–brain barrier. J Cereb Blood Flow Metab. 2018:0271678X1878337. https://www.ncbi.nlm.nih.gov/pubmed/29911470. 10.1177/0271678X18783372 PMC6827128 29911470

[15] Chiou B, Neal EH, Bowman AB, Lippmann ES, Simpson IA, Connor JR (2018). Pharmaceutical iron formulations do not cross a model of the human blood-brain barrier. PLoS One.

[16] Liu H, Xu E, Liu J, Xiong H (2016). Oligodendrocyte Injury and Pathogenesis of HIV-1-Associated Neurocognitive Disorders. Brain Sci.

[17] Heaton RK, Clifford DB, Franklin DR, Woods SP, Ake C, Vaida F (2010). HIV-associated neurocognitive disorders persist in the era of potent antiretroviral therapy: CHARTER Study. Neurology.

[18] Heaton RK, Franklin DR, Ellis RJ, McCutchan JA, Letendre SL, LeBlanc S (2011). HIV-associated neurocognitive disorders before and during the era of combination antiretroviral therapy: differences in rates, nature, and predictors. J Neurovirol.

[19] Jernigan TL, Archibald SL, Fennema-Notestine C, Taylor MJ, Theilmann RJ, Julaton MD (2011). Clinical factors related to brain structure in HIV: the CHARTER study. J Neurovirol.

[20] Fennema-Notestine C, Ellis RJ, Archibald SL, Jernigan TL, Letendre SL, Notestine RJ (2013). Increases in brain white matter abnormalities and subcortical gray matter are linked to CD4 recovery in HIV infection. J Neurovirol.

[21] Koda T, Namba A, Nakatsuji Y, Niino M, Miyazaki Y, Sugimoto T (2018). Beneficial effects of fingolimod in MS patients with high serum Sema4A levels. PLoS One.

[22] Connor JR, Ponnuru P, Wang X-S, Patton SM, Allen RP, Earley CJ (2011). Profile of altered brain iron acquisition in restless legs syndrome. Brain.

[23] Nandar W, Neely EB, Simmons Z, Connor JR (2014). H63D HFE genotype accelerates disease progression in animal models of amyotrophic lateral sclerosis. Biochim Biophys Acta - Mol Basis Dis.

[24] Lu N, Li Y, Zhang Z, Xing J, Sun Y, Yao S (2018). Human Semaphorin-4A drives Th2 responses by binding to receptor ILT-4. Nat Commun..

[25] Ishii H, Kubo T, Kumanogoh A, Yamashita T (2010). Th1 cells promote neurite outgrowth from cortical neurons via a mechanism dependent on semaphorins. Biochem Biophys Res Commun.

[26] Hubler Z, Allimuthu D, Bederman I, Elitt MS, Madhavan M, Allan KC (2018). Accumulation of 8,9-unsaturated sterols drives oligodendrocyte formation and remyelination. Nature.

[27] Mangale V, McIntyre LL, Walsh CM, Loring JF, Lane TE. Promoting remyelination through cell transplantation therapies in a model of viral-induced neurodegenerative disease. Dev Dyn. 2018;248(1):43–52. 10.1002/dvdy.24658 PMC6420815 30067309

[28] Nkyimbeng-Takwi E, Chapoval SP (2011). Biology and function of neuroimmune semaphorins 4A and 4D. Immunol Res.

[29] Leitner DF, Stoute JA, Landmesser M, Neely E, Connor JR (2015). The HFE genotype and a formulated diet controlling for iron status attenuate experimental cerebral malaria in mice. Int J Parasitol.

[30] Ragin AB, Wu Y, Gao Y, Keating S, Du H, Sammet C (2015). Brain alterations within the first 100 days of HIV infection. Ann Clin Transl Neurol.

[31] Liuzzi GM, Mastoianni CM, Fanelli M, Massetti AP, Vullo V, Delia S (1994). Myelin degrading activity in the CSF of HIV-1-infected patients with neurological diseases. Neuroreport.

[32] Del Valle L, Piña-Oviedo S (2006). HIV disorders of the brain: pathology and pathogenesis. Front Biosci.

[33] Gongvatana A, Schweinsburg BC, Taylor MJ, Theilmann RJ, Letendre SL, Alhassoon OM (2009). White matter tract injury and cognitive impairment in human immunodeficiency virus–infected individuals. J Neurovirol.

[34] Cysique LA, Soares JR, Geng G, Scarpetta M, Moffat K, Green M (2017). White matter measures are near normal in controlled HIV infection except in those with cognitive impairment and longer HIV duration. J Neurovirol.

[35] Gold J, Goldacre R, Maruszak H, Giovannoni G, Yeates D, Goldacre M (2015). HIV and lower risk of multiple sclerosis: beginning to unravel a mystery using a record-linked database study. J Neurol Neurosurg Psychiatry.

[36] Kumanogoh A, Kikutani H (2003). Immune semaphorins: a new area of semaphorin research. J Cell Sci.

[37] Chabbert-de Ponnat I, Marie-Cardine A, Pasterkamp RJ, Schiavon V, Tamagnone L, Thomasset N (2005). Soluble CD100 functions on human monocytes and immature dendritic cells require plexin C1 and plexin B1, respectively. Int Immunol.

